# The lifetime risk of surgery in England: a nationwide observational cohort study

**DOI:** 10.1016/j.bja.2024.06.028

**Published:** 2024-07-31

**Authors:** Sarah-Louise Watson, Alexander J. Fowler, Priyanthi Dias, Bruce Biccard, Yize I. Wan, Rupert M. Pearse, Tom E.F. Abbott

**Affiliations:** 1 Barts and the London School of Medicine and Dentistry, Queen Mary University of London, London, UK; 2 Critical Care and Perioperative Medicine Research Group, William Harvey Research Institute, Queen Mary University of London, London, UK; 3 Department of Anaesthesia and Perioperative Medicine, Groote Schuur Hospital, Faculty of Health Sciences, University of Cape Town, Cape Town, South Africa

**Keywords:** COVID-19, cumulative incidence, epidemiology, health services research, life tables, lifetime risk, surgery

## Abstract

**Background:**

The average number of times a person will have surgery in their lifetime, and the amount of surgical healthcare resources they use, is unknown. Lifetime risk is a measure of the risk of an average person having a specific event within their lifetime. We report the lifetime risk of surgery and the change observed during the first year of the COVID-19 pandemic.

**Methods:**

We conducted a population cohort study using hospital episode statistics to identify all patients undergoing surgery between January 1, 2016, and December 31, 2020, in England. We calculated age- and sex-specific incidence rates of surgery and combined these with routinely available population and mortality data from the Office for National Statistics. We computed the probability of requiring surgery stratified by 5-yr epochs (age 0–4 to ≥90 yr). Our primary analysis calculated lifetime risk for all surgery using the life table method. We assessed the impact of the COVID-19 pandemic, comparing a pre-pandemic and a pandemic period.

**Results:**

Between 2016 and 2020, 23 427 531 patients underwent surgery, of which 11 937 062 were first surgeries. The average denominator population for England was 55.9 million. The lifetime risk of first surgery was 60.2% (95% confidence interval 55.1–65.4%) for women and 59.1% (95% confidence interval 54.2–64.1%) for men. The COVID-19 pandemic decreased the lifetime risk of first surgery by 32.3% for women and by 31.7% for men. This estimated lifetime risk should only be applied to the English population.

**Conclusions:**

This population epidemiological analysis suggests that approximately 60% of people in England will undergo surgery in their lifetime.

**Editor's key points**

• Lifetime risk of surgery and the change observed during the first year of the COVID-19 pandemic have not been reported.
• The authors conducted a population cohort study using hospital episode statistics to identify all patients undergoing surgery between 2016 and 2020 in England to determine age- and sex-specific incidence rates of surgery.
• The lifetime risk of first surgery was ∼60% for women and men. The COVID-19 pandemic decreased the lifetime risk of first surgery by ∼30% for women and men.
• Lifetime risk of surgery is a useful public health statistic for understanding the burden of surgery and provides patients with an estimate of their risk of surgery in their lifetime.

Surgery represents a large portion of UK National Health Service (NHS) activity; each year, ∼4.4 million people undergo surgical procedures in the NHS in England. Although, increasingly, surgery is performed as day-case procedures, a substantial portion of surgical activity continues to require an overnight stay in the hospital, with a median length of stay of 1.7 days.^1^ Notably, one in six patients develop postoperative complications. These vary in severity and can lead to increased hospital length of stay and resource use.^2^ Moreover, postoperative complications experienced within 30 days following surgery cause a 1-yr mortality rate to increase by two-fold.^3^ The surgical population is ageing; patients undergoing surgery are, on average, 14.5 yr older than the general population.^4^ In England, 20% of the population aged 75 yr and older underwent surgery in 2015, and this number is increasing.^4^ Older age is associated with poorer postoperative outcomes, potentially resulting in greater use of healthcare resources.^4^ The recent COVID-19 pandemic also influenced how we organise healthcare systems and thus surgical trends and volume. It is not known how many times the average person will have surgery in their lifetime and thus one aspect of their healthcare use.

Lifetime risk is a measure of the risk of an event occurring over an average person's lifetime. It is commonly used to calculate the risk of developing certain diseases, for example cancer or diabetes mellitus, but it has not previously been used to quantify exposure to surgical procedures.^5^, ^6^, ^7^ When considering the epidemiology of non-communicable diseases, such as cancer, quantifying individual risk of developing a disease is important for public health and healthcare resource planning. Knowledge of the lifetime risk of undergoing surgery supports public health strategists' planning of resource and workforce requirements across the healthcare system.

The COVID-19 pandemic had a substantial impact on elective surgical services. Global estimations suggested that 72.3% of all operations would be cancelled or postponed during the 12-week peak of COVID-19.^8^ In the NHS in England and Wales, there was a 33.6% decrease in surgical volume, representing cancellation of more than 1.5 million surgeries in 2020 alone.^9^ For the average member of the population, it is unclear how the pandemic has impacted the risk of having surgery across their lifetime. We aimed to calculate the lifetime risk of undergoing surgery in England and report any change to this as a result of the COVID-19 pandemic.

## Methods

### Study design

We conducted a population cohort study using Hospital Episode Statistics for Admitted Patient Care (HES-APC) including all patients undergoing surgery in the NHS in England between January 1, 2016, and December 31, 2020. We sourced population and mortality estimates of England for 5-yr epochs from aged 0–4 to ≥90 yr using data from the Office of National Statistics (ONS) (Newport, UK).

### Data source

We used HES-APC for England to calculate the number of surgeries occurring in each age interval and the 30-day mortality rate of surgery in the defined period. HES is a database encompassing data on all admitted patient care, outpatient attendances, and urgent and emergency care activities in England. This analysis of routinely collected, pseudonymised data was approved by the Health Research Authority (20/HRA/3121). Access to NHS England data was approved by the NHS Digital Independent Group Advising on the Release of Data (DARS–NIC–375669-J7M7F).

HES-APC specifically gathers information regarding all patient hospital admissions. This includes data on diagnoses, operations, patient characteristic information, dates of admission, and discharge and death data from ONS civil registry data.^10^ HES-APC uses Office of Population Census and Surveys Version 4 (OPCS-4) codes to define any intervention or procedure patients have during their hospital stay. We used the ONS to gather data on the age- and sex-specific size of the population and all-cause mortality. This information is released annually.

### Participants

The denominator population is the annual general population of England for each year of data recorded, which we sourced using population data from the ONS. We identified all patients, of any age, undergoing surgery using HES-APC in England between January 1, 2016, and December 31, 2020. Surgery was defined using a previously published list of OPCS-4 codes, which represent individual surgical procedures.^1^

### Outcomes

The primary outcome measure was the number of patients undergoing a surgical procedure defined by the predetermined list of OPCS-4 codes.

### Statistical methods

This analysis was pre-planned using a prospective statistical analysis plan (Supplementary Appendix S1). We reported the total number of patients undergoing surgery defined by the OPCS-4 codes in the time frame assessed. We divided the data into 5-yr epochs from age 0–4 to ≥90 yr and stratified the data by sex. To calculate the incidence rate of surgery per head of the population, we divided the total number of patients undergoing surgery for each 5-yr epoch by the number of person-years in each 5-yr epoch.

There are two validated methods to measure lifetime risk: life tables and cumulative incidence.^11^ Life tables uses an approach considering both mortality and incidence rates, whereas cumulative incidence uses an alternate approach considering only incidence rates. We used life tables as our primary approach but repeated the analysis using cumulative incidence. In Supplementary Appendix S2, we provide the formulae used to calculate lifetime risk. Both were calculated using data from January 1, 2016 to December 31, 2019, as this does not include the pandemic period. For each method we provide a 95% confidence interval (CI). We calculated the lifetime risk of surgery for 2016 and repeated this calculation for consecutive years for which data were available, using an approach from Ahmad and colleagues.^5^

### Statistical analysis

We used Microsoft Excel (Redmond, WA, USA) for data processing. We calculated 95% CIs from the average lifetime risk of surgery, using the life table method, from 2016 to 2019 for the pre-pandemic period. This gave upper and lower 95% CIs. We repeated this for cumulative incidence. Our defined pandemic period was 1 yr; therefore, we could not average the lifetime risk of surgery across multiple years. We calculated the 95% CI of the lifetime risk of surgery in 2020.

### Derivation of variables

Age was defined as the age of the patient on the date of their operative procedure. HES includes data on all surgical procedures; thus, it is possible for one patient to undergo more than one surgical procedure. Surgical procedures are defined as procedures routinely undertaken in an operating theatre, under general or regional anaesthesia, or both, according to a published list of OPCS-4 codes.^1^ We calculated the lifetime risk of someone undergoing any surgery and a first surgery. First surgery is the first surgery documented in the HES record based on date. All surgery was defined as all operations taking place within NHS England that year, including people who underwent multiple surgeries on different dates.

### Impact of COVID-19 on the lifetime risk of surgery

To assess the impact of COVID-19 on the lifetime risk of surgery, we analysed a pre-pandemic period and a pandemic period. We defined the pre-pandemic period as January 1, 2016 to December 31, 2019, and the pandemic period as January 1, 2020 to December 31, 2020. We calculated the lifetime risk for the pre-pandemic period using an average of the lifetime risks calculated using the life table method from 2016 to 2019 and repeated this for cumulative incidence. We compared the pandemic period with the pre-pandemic period by assessing overlap in the 95% CIs and using a paired *t*-test. A *P*-value of 0.05 was considered statistically significant.

### Sensitivity analysis

We undertook a *post hoc* sensitivity analysis, which excluded patients undergoing obstetric procedures, and repeated the analysis. This was to investigate the influence of obstetric operations on the lifetime risk of surgery.

## Results

### Participants

**Fig 1:**
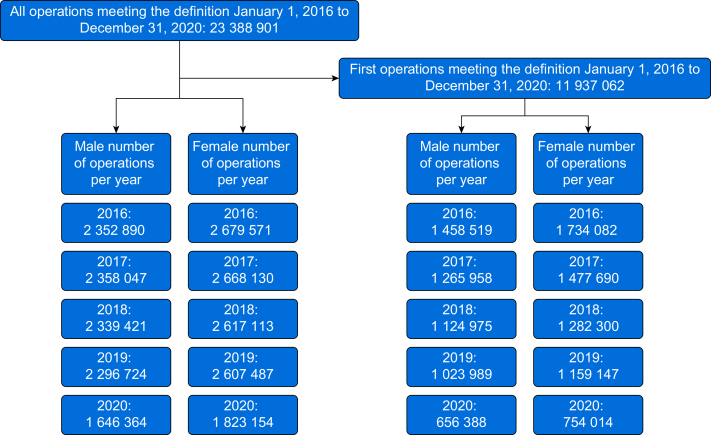
Flow chart summarising inclusion of patients in the analysis.

There were 23 427 531 surgical procedures and 11 937 062 first surgeries that took place between January 1, 2016 and December 31, 2020; the participants included are shown in Figure 1. The average total denominator population for England was 55.9 million.

### Lifetime risk of surgery

#### Lifetime risk calculated using the life table method

The lifetime risk of first surgery was 60.2% (95% CI 55.1–65.4%) for women and 59.1% (95% CI 54.2–64.1%) for men, and the lifetime risk of all surgery was 80% (95% CI 78.6–81.4%) for men and 79.5% (95% CI 78.1–80.9%) for women (Table 1).

**Table 1:** Lifetime risk of all surgery and first surgery for men and women based on the years 2016–2019. This is split by age- and sex-specific epochs. Each was calculated to a 95% CI shown in brackets. CI, confidence interval.

	Men		Women	
Age (yr)	Lifetime risk of first surgery, % (95% CI)	Lifetime risk of all surgery, % (95% CI)	Lifetime risk of first surgery, % (95% CI)	Lifetime risk of all surgery, % (95% CI)
0–4	3.1 (2.9–3.3)	4.5 (4.5–4.6)	2.1 (1.9–2.2)	3.1 (3–3.1)
5–9	5.1 (4.7–5.6)	7.3 (7.1–7.5)	3.6 (3.3–3.9)	5.2 (5–5.3)
10–14	7.1 (6.5–7.8)	10 (9.7–10.3)	5.1 (4.7–5.6)	7.3 (7.1–7.5)
15–19	9.4 (8.6–10.2)	13 (12.7–13.3)	7.4 (6.8–8)	10.3 (10–10.6)
20–24	11.8 (10.8–12.8)	16.2 (15.8–16.6)	10.8 (9.8–11.9)	15.1 (14.6–15.5)
25–29	14.3 (13–15.5)	19.6 (19.1–20.1)	15.4 (13.8–16.9)	21.3 (20.7–21.8)
30–34	16.8 (15.4–18.2)	23.1 (22.5–23.6)	20.2 (18.2–22.3)	27.9 (27.6–28.4)
35–39	19.4 (17.7–20.9)	26.7 (26.1–27.3)	24.3 (21.8–26.9)	33.6 (32.9–34.2)
40–44	22 (20.2–23.9)	30.5 (29.8–31.2)	27.5 (24.6–30.4)	38.2 (37.5–38.9)
45–49	24.9 (22.8–27)	34.8 (34–35.6)	30.6 (27.3–33.9)	42.8 (42–43.6)
50–54	28.1 (25.6–30.6)	39.6 (38.8–40.5)	33.7 (30.1–37.3)	47.5 (46.6–48.3)
55–59	31.7 (28.9–34.5)	45.2 (44.4–46.1)	36.9 (33–40.8)	52.1 (51.2–53)
60–64	35.8 (32.6–39)	51.5 (50.6–52.3)	40.3 (36.1–44.4)	56.9 (56–57.8)
65–69	40.2 (36.7–43.8)	58.1 (57.1–59)	43.9 (39.5–48.3)	61.9 (60.9–62.8)
70–74	44.8 (40.8–48.8)	64.5 (63.5–65.6)	47.8 (43.1–52.5)	66.9 (65.8–67.9)
75–79	49.4 (45–53.8)	70.5 (69.3–71.6)	51.8 (46.9–56.7)	71.6 (70.4–72.8)
80–84	53.5 (48.9–58.2)	75.1 (73.9–76.4)	55.4 (50.4–60.4)	75.4 (74.1–76.7)
85–89	56.9 (52–61.8)	78.3 (77–79.7)	58.3 (53.2–63.4)	78.1 (76.7–79.5)
≥90	59.1 (54.2–64.1)	80 (78.6–81.4)	60.2 (55.1–65.4)	79.5 (78.1–80.9)

**Fig 2:**
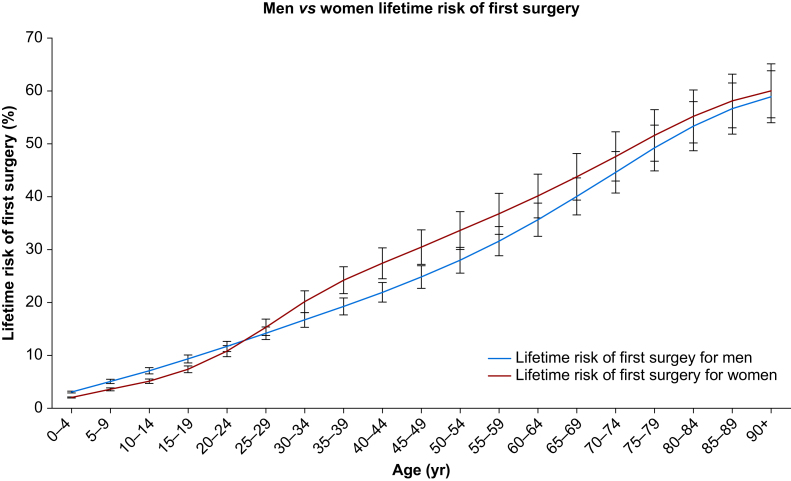
Men (blue line) compared with women (red line) lifetime risk of first surgery, calculated using the life table method, for the years 2016–2019. Error bars show the 95% confidence intervals.

**Fig 3:**
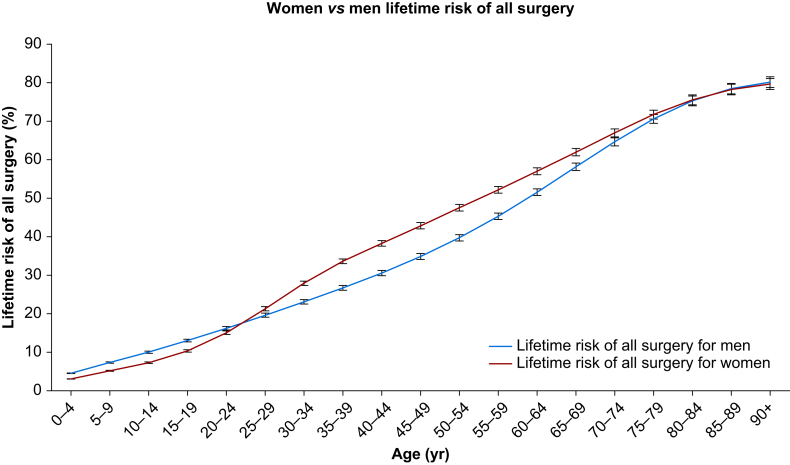
Men (blue line) compared with women (red line) lifetime risk of all surgery calculated for the years 2016–2019. Error bars show the 95% confidence intervals.

Women have a higher lifetime risk of first surgery (Fig. 2). This trend starts from ages 25–29 yr, with the difference between men and women increasing until ages 45–49 yr. The largest increase in lifetime risk of first surgery between age ranges occurs at 25–29 to 30–34 yr for women, with a 4.9% increase, and at 70–74 to 75–79 yr for men, with a 4.6% increase. Figure 3 shows that men have an overall higher lifetime risk of all surgery. However, between ages 25–29 and 65–69 yr, women have a higher lifetime risk of all surgery. The difference between the sexes peaks at ages 45–49 yr, where women have an 8% increase in risk. The largest increases in lifetime risk of all surgery between age ranges occur at 25–29 to 30–34 yr for women, with a 6.6% increase, and at 60–64 to 65–69 yr for men, with a 6.6% increase.

**Fig 4:**
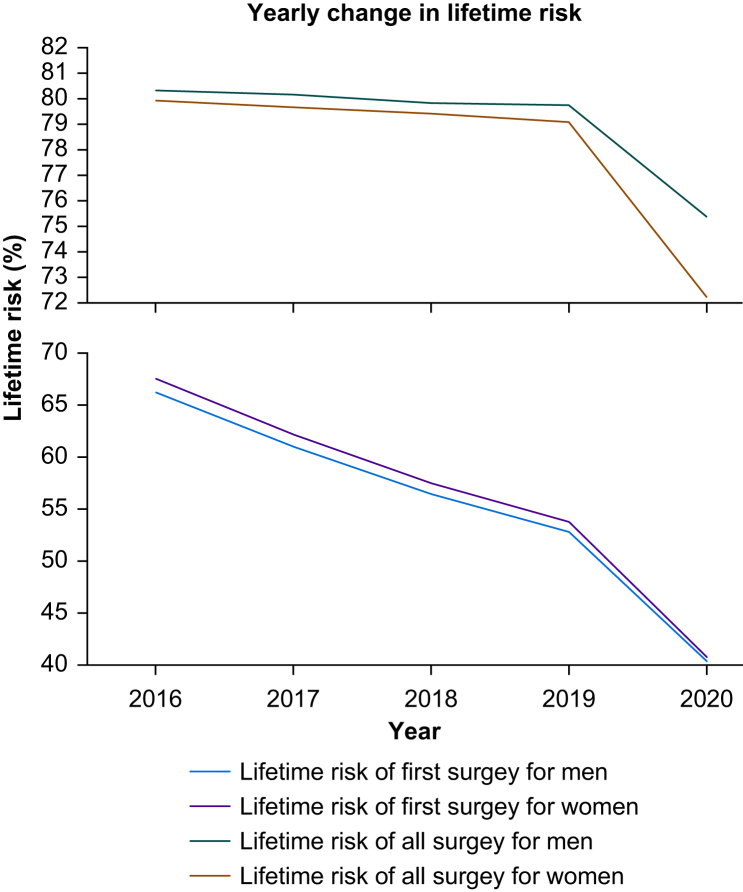
Yearly change in lifetime risk for all and first surgery, calculated using the life table method, from 2016 to 2020.

The lifetime risk of first surgery declined each year between 2016 and 2019 (Fig. 4). The biggest decline occurred between 2016 and 2017, with a 5% decrease for men and women. There was a negligible decline each year between 2016 and 2019 for all surgery.

#### Cumulative incidence of surgery

The cumulative lifetime incidence of first surgery was 64.4% (95% CI 57.9–70%), and the cumulative incidence of all surgery was 87.7% (95% CI 87.3–88.2%). When dividing the data by sex, the cumulative incidence of first surgery was 64.9% (95% CI 58.3–70.5%) for women and 64.4% (95% CI 57.7–70%) for men (Supplementary Fig. S1). The cumulative incidence of all surgeries was 88.9% (95% CI 88.5–89.3%) for men and 87.1% (95% CI 86.6–87.7%) for women (Supplementary Fig. S2). Women had a higher cumulative incidence of first surgery, and men had a higher cumulative incidence of all surgery.

### Impact of COVID-19

The lifetime risk of first surgery decreased from 60.2% (95% CI 55.1–65.4%) to 40.8% (95% CI 39.3–42.2%) in women and 59.1% (95% CI 54.2–4.1%) to 40.4% (95% CI 38.8–41.9%) in men (Supplementary Figs. 3 and 4). The lifetime risk of all surgery decreased from 79.5% (95% CI 78.1–80.9%) to 72.2% (95% CI 70.8–73.7%) in women and 80% (95% CI 78.6–81.4%) to 75.4% (95% CI 73.8–77%) in men (Supplementary Figs. 3 and 4). The lifetime risk calculated using both methods for all surgery and first surgery decreased significantly (*P*<0.05) during the pandemic.

### Sensitivity analysis

In a sensitivity analysis removing obstetric operations, the lifetime risk of first surgery without obstetric procedures was 57.8% (95% CI: 52.6–62.9) for women and 58.7% (95% CI: 53.8–63.6) for men, and the lifetime risk of all surgery was 78% (95% CI: 76.5–79.4) for women and 79.5% (95% CI: 78.1–80.9) for men.

## Discussion

The principal finding of this study is that the lifetime risk of having surgery is ∼60%. For a woman born between 2016 and 2019 living ≥90 yr, they would have a 60.2% chance of undergoing surgery in their lifetime if the incidence and mortality rates remain constant.

It is important to consider the impact on our health service in terms of workforce requirements and resources required to meet this demand. Previous research has not extensively explored the lifetime risk of surgery; instead, studies have focused on the lifetime risk of specific surgeries or surgery related to a specific disease.^12^^,^^13^ A previous study measured the lifetime risk of surgery in three US states calculated by summing the rates of surgical procedures for each year of ages between 0 and 84 yr.^14^ The average person underwent 5.97 surgical procedures throughout an 85-yr lifespan.^14^ This is not comparable to our study as we used a different methodology. Furthermore, differences in surgical procedures included in each study and regional variations in surgical utilisation would alter lifetime risk.^15^ When specifically comparing the methods used with those of previous studies, our study found that cumulative incidence predicted a higher lifetime risk of surgery than the life table method, in line with earlier studies.^5^^,^^16^ Cumulative incidence does not consider the competing risk of mortality, causing an overestimation of lifetime risk.^11^

We calculated a larger difference in lifetime risk between methods for men. This might be explained by higher mortality rates and lower life expectancy of men (78.6 yr) in comparison with women (82.6 yr). This mortality difference would be reflected in the life table method and lead to a larger difference between methods. Women had a greater lifetime risk of all surgery between 25–29 and 65–69 yr in comparison with men. This could be explained by obstetric and gynaecological surgery. When we repeated the analysis after removing obstetric operations, women only have a slightly increased lifetime risk than men between ages 30–34 and 65–69 yr. When obstetric operations are removed, women have a lower lifetime risk of first surgery than men. There was a small decline in the number of operations for men, which mainly occurred in the age range 0–4 yr. This is because infants born during an episode are judged to have arrived during a maternity episode, and owing to HES coding, they were removed with the obstetrics procedures. Some differences could reflect a wider variation in utilisation of healthcare between men and women. For example, there are differences in consultation rates, with men less likely to attend primary care consultations, differences between a surgical or conservative approach, and differences in the prevalence of conditions requiring surgery.^17^^,^^18^ We did not assess association of other factors linked to health inequality, including social deprivation and ethnicity. These factors influence access to healthcare, patient health and outcomes, and mortality rates and would likely impact lifetime risk of surgery.^19^, ^20^, ^21^, ^22^

The lifetime risk during the pandemic period decreased by 9% for women and 6% for men for all surgery and by 32% for women and 31% for men for first surgery. There was a 33.6% decrease in the volume of surgery during the pandemic.^23^ This was due to multiple factors including redeployment of the workforce, exposure to COVID-19, and added infection control procedures.^24^^,^^25^ Furthermore, the pandemic resulted in 139 000 excess deaths causing the all-cause mortality rate in 2020 to increase to levels previously seen a decade ago.^26^^,^^27^ Both the increase in all-cause mortality rate and the decrease in volume of surgery explain the decrease in lifetime risk of surgery.^9^

This study has several strengths. Lifetime risk is a validated and understandable statistic used in multiple areas of medicine including cancer and cardiovascular disease.^16^^,^^28^^,^^29^ The methods were validated and used in previous studies.^5^ The study uses HES-APC statistics to calculate the number of surgeries taking place in the NHS for each age group. This represents any surgery undertaken by, or paid for by, NHS England. The all-cause deaths and population data were taken from the ONS, which uses high-quality administrative sources to report birth and death registration.^30^ There are also limitations to this study. Our calculation for the total number of surgeries might be an underestimation. HES relies on hospital workers to enter OPCS-4 codes for each patient episode, so human error could mean coding mistakes are made.^1^ When multiple procedures happen in one hospital episode, only the predominant procedure is recorded.^1^ Moreover, HES does not include surgery taking place in the private sector, unless funded by NHS England.

Before the pandemic, 93.6% of elective care was NHS funded and 6.4% was privately funded and therefore not included in our analysis.^31^ During the first year of the pandemic, 6.3% was privately funded and thus not included.^31^ There could be further bias related to the devolved administrations (e.g. Scotland, Wales, or Northern Ireland). If a patient resides close to the border, they might receive emergency surgery in England despite living in a devolved administration and *vice versa*. Furthermore, some surgeries might be provided for by NHS England for the devolved administrations.

Regarding the COVID-19 pandemic impact, we defined the pandemic period from January 1 to December 31, 2020. This differs from when the WHO declared a pandemic on March 11, 2020, meaning we recorded an additional 70 days of data where surgical activity was normal.^32^ We could not calculate the pandemic period from March as data regarding the number of surgeries were given annually, not monthly, underestimating the impact of the pandemic. Furthermore, the study pandemic period ended on December 31, 2020 when our data collection ended, but the pandemic continued, further impacting surgical volume and lifetime risk of surgery.^33^ We were also unable to investigate any rebound effect caused by the pandemic.

There are limitations to the methods used to measure lifetime risk. The most accurate way to measure lifetime risk would be to undertake a population-wide cohort study lasting a full lifetime. However, this is not feasible on resource and economic grounds. Thus, we adopted a previously validated methodology to estimate lifetime risk. Both cumulative lifetime incidence and the life table method are affected by changing incidence and mortality rates. Our data cannot be applied to a population with different incidence and mortality rates and is premised on circumstances staying the same as the observed period, which is unlikely. Furthermore, changes to life expectancy and longevity of the overall population will impact survival, altering lifetime risk. The lifetime risk statistic is also population data, meaning it will not apply to an individual more likely to undergo surgery than the average population.

### Conclusions

In England during our study period, 60% of people underwent surgery in their lifetime. We calculated this using the life table method, which considers the competing risk of mortality. The COVID-19 pandemic significantly decreased lifetime risk of all surgery and first surgery. Lifetime risk of surgery is a useful statistic in helping public health strategists understand the burden of surgery. It also provides the public with a useful statistic on the risk of surgery throughout their lifetime.

## Authors’ contributions

Design: SLW, AJF, YW, RP, TA

Analysis and interpretation of data: SLW, AJF, TA

Writing the first draft of manuscript: SLW, TA

Concept: AJF, BB, TA

Reviewing and approving the final draft of the manuscript: PD, BB, YW, RP

Study guarantor: TA

Critical revision of manuscript for important intellectual content and approval of the final version of manuscript: all authors

## Acknowledgements

The funding sources had no role in the study design, data collection, analysis, interepretation of data or writng the manuscript. The authors acknowledge and thank all those involved in the collection of data.

## Declarations of interest

This research was not pre-registered, but a statistical analysis plan was developed before starting the study, as shown in Supplementary Appendix S1. TA is supported by an NIHR clinical lectureship; has received research funding from Barts Charity, the Academy of Medical Sciences, The Royal College of Anaesthetists, and the *British Journal of Anaesthesia*; has received honoraria from MSD and Edwards Life Sciences; and is Social Media Editor of the *British Journal of Anaesthesia*. AJF has received funding from NIHR DRF 2018-11-ST2-062. RMP has received honoraria, research grants, or both from Edwards Lifesciences, Intersurgical, and GlaxoSmithKline within the past 5 yr and holds editorial roles with the *British Journal of Anaesthesia* and the *British Journal of Surgery*.

## Funding

Grant from Barts Charity (MGU0545) to TA.
