# Supplementary material for: The lifetime risk of surgery in England: a nationwide observational cohort study
Source: Br J Anaesth. 2024 Jul 31;133(4):768–75. doi: 10.1016/j.bja.2024.06.028 (PMC11443128; doi:10.1016/j.bja.2024.06.028)
Supplement: Multimedia component 1 [file mmc1.docx]

**Supplementary data**

**Appendix 1**

Statistical analysis plan

**Population lifetime risk of surgery and the impact of the COVID-19 pandemic: A Nationwide observational cohort study**

**Statistical Analysis Plan**

**3^rd^ January 2023**

**Authors:** Watson, Biccard, Fowler, Dias, Wan, Pearse, Abbott

Version 0.9

1. **Introduction**

***Background and rationale***

Each year approximately 5.1 million people undergo surgery representing a large proportion of NHS activity(1). Complications arise in one in five surgical procedures, which leads to prolonged hospital stays and higher costs associated with surgery and staying in hospital(2). The 30-day mortality rate of surgery is 1%, but one-year mortality rates increase by two-fold if a patient experiences a complication within the first 30 days after surgery(1, 2). Currently it is unknown how many times the average person will have surgery during their lifetime and thus how much surgical healthcare resources the average person will use over their lifetime.

Lifetime risk is a measure of the risk of an event occurring throughout an average person’s lifetime. It is a measurement that is used commonly to calculate a patient’s risk of developing a certain disease, for example breast cancer or cardiovascular disease, but it has not been used to assess the risk of a patient undergoing any type of surgery during their lifetime(3, 4). Knowing a patient’s lifetime risk of undergoing surgery could potentially help public health strategists anticipate and plan for the future burden of surgery, potential complications and mortality. Ideally, to calculate the lifetime risk of a patient undergoing surgery we would use a cohort study to assess a population across their lifetime and investigate if and at what age participants undergo surgery and when the participants die. However, this has limited feasibility at a population level. Instead, we will use two validated methods to calculate the lifetime risk of surgery: life tables and cumulative risk.

The COVID-19 pandemic has changed how healthcare is delivered and led to large changes in resource allocation(5). The pandemic has had a clear impact on surgery and waiting lists. However, the magnitude of this has not yet been quantified(6). This unmeasured impact could have influenced the lifetime risk of undergoing surgery during the pandemic period. We will use COVID-19 as an exposure to investigate the impact it has had on the lifetime risk of surgery.

This study aims to measure the lifetime risk of undergoing surgery and report any change in the lifetime risk during the COVID-19 pandemic.

***Primary Objective:***

1. To report the population lifetime risk of surgery.

***Secondary Objective:***

1. To report the impact of the COVID-19 pandemic on the lifetime risk of surgery.
2. **Methods**

***Data source***

The study will be conducted in England. Data regarding the number of surgeries taking place will be sourced from hospital episode statistics admitted patient care (HES-APC). This database gathers information regarding all patient hospital admissions. This includes data on clinical information regarding diagnoses and operations, patient demographic information and dates of admission and discharge(7). Data on population estimates will be sourced from life tables produced by the ONS(8).

***Study design and setting***

This is a nationwide observational cohort study that will describe the number of patients undergoing surgery in aged-specific 5-year intervals in the National Health Service (NHS) in England between 1^st^ April 2015 and 31^st^ August 2020_._ This will then be compared with data on population estimates from the ONS for each 5-year age interval to calculate lifetime risk. The impact of the COVID-19 pandemic on the lifetime risk of surgery will be assessed by comparing data from a pre-pandemic period defined as __ to the ____,_ to a pandemic period defined as __ to the ____._

***Participants and data collection***

All patients, of any age, identified as undergoing surgery using HES-APC in England between 1^st^ April 2015 and 31^st^ August 2020 will be included. Surgery will be defined using the assigned Office of Population Census and Surveys Version 4 (OPCS-4) codes for hospital procedures. Surgical procedures will be identified by using a predetermined list of OPCS-4 codes(1). The number of patients undergoing surgery will be categorised into 5-year age bands. Population estimates will be found using national life tables released from the ONS, this again will be categorised into aged-specific 5-year age bands(8).

***Derivation of variables***

Age will be defined by the age that the patient was on the date of their operative procedure. The number of patients undergoing surgery will be categorised into 5-year age epochs e.g. Age 0-4, 5-9, 10-14…. until 90+. The lifetime risk will be calculated and repeated for consecutive years that we have data available, this has been done in previous papers assessing the lifetime risk of diabetes(9, 10).

***Outcomes***

*Primary outcome*

1. A surgical procedure defined according to a standard definition.

*Secondary outcome*

1. Death among patients who have had surgery.
2. **Statistical analysis**

***Statistical Analysis***

There are two validated methods to measure lifetime risk – life tables and cumulative risk. We will calculate the lifetime risk of surgery using both methods, with life table’s being the primary method.

We will report the total number of patients undergoing surgery, as defined using the OPCS-4 codes, in the time frame assessed. This data will be categorised by 5-year epochs. We will then divide the total number of patients undergoing surgery for each 5-year epoch by the total population in that 5-year epoch using population estimates from ONS data. This will give us the age-specific incidence rates of surgery per head of the population.

**Life tables**

We will report the lifetime risk of surgery using life tables, this is considered a more accurate method for lifetime risk in comparison with cumulative risk as it considers the competing risk of death in the population(11). This will be calculated with a 95% confidence interval. The lifetables method has been used to calculate the lifetime risk of diabetes(9, 10). To calculate lifetime risk via the lifetable method we need to find the incident rate, mortality rate of those that have had surgery and the mortality rate for those who have not had surgery in each 5-year epoch. This can be done using the following equations(9):


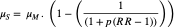


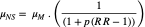


Where
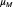
 is all-cause mortality,
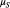
 is the age-specific morality rate for those who have had surgery,
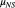
 is the age-specific morality rates for those that have not had surgery, Relative risk (RR) is the age-specific relative risk of mortality for those who have had surgery and
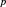
 is the age-specific prevalence of surgery.

The incident and mortality rates can then be used to find out the probability that a patient will transition from the state of not having surgery to having surgery or death and from having surgery to death, this is shown in the following equations(9):

Probability of transitioning from no surgery to death:


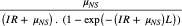


Probability of transitioning from no surgery to surgery:


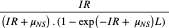


Probability of transitioning from surgery to death:


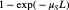


Where IR is the age-specific incident rate of surgery and L is a period of time (five years). This will allow us to calculate the probability that a person aged x who has not had surgery will have surgery before reaching age x+1 or will die without having surgery before reaching age x+1. This can be applied to a hypothetical cohort to estimate the number of individuals in the population undergoing surgery at each 5-year epoch.

**Cumulative risk**

Cumulative risk can be used to estimate the lifetime risk of having surgery. This is calculated using the following equation(11):


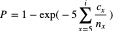


Where P is the cumulative risk until age
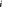
, C_x_ is the total number of surgeries in age group
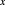
 and n_x_ is the number of person-years of age group
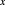
. This is multiplied by 5 as 5-year epochs are used. This will be calculated with a 95% confidence interval.

***Impact of COVID-19 Pandemic***

We will consider COVID-19 as an exposure and calculate its impact on the lifetime risk of having surgery. The impact of the COVID-19 pandemic on the lifetime risk of surgery will be investigated by assessing for any difference in lifetime risk in the specified time-period for pre-pandemic and pandemic. We will do this by generating the five-year epochs for the pre-pandemic period and then for the pandemic period and comparing the two. To investigate for a significant statistical difference between the lifetime risk of surgery in the pre-pandemic period and the pandemic period we will use two methods. The first test will involve using 95% confidence intervals(12). If the 95% confidence intervals for the lifetime risk of surgery in the pre-pandemic period does not overlap with the 95% confidence intervals in the pandemic period or the 95% uncertainty interval for relative percentage change does not include 0 then there will be a statistically significant difference in the lifetime risk of surgery between the pre-pandemic and pandemic periods(12). The second test will be a T-test, a P-value 0.05 will be considered statistically significant.

***Research Ethics approval***

This analysis of routinely collected, pseudonymised data was approved by the Health Research Authority (20/HRA/3121). Access to NHS England data was approved by the NHS Digital Independent Group Advising on the Release of Data (DARS-NIC-375669-J7M7F).

***Handling of missing data***

Patients missing data will be excluded from the analysis.

**References**

[1. Abbott TEF, Fowler AJ, Dobbs TD, Harrison EM, Gillies MA, Pearse RM. Frequency of surgical treatment and related hospital procedures in the UK: a national ecological study using hospital episode statistics. Br J Anaesth. 2017;119(2):249-57.](https://digital.nhs.uk/data-and-information/publications/statistical/hospital-admitted-patient-care-activity/2020-21)

[2. Fowler AJ, Wan YI, Prowle JR, Chew M, Campbell D, Cuthbertson B, et al. Long-term mortality following complications after elective surgery: a secondary analysis of pooled data from two prospective cohort studies. Br J Anaesth. 2022;129(4):588-97.](https://digital.nhs.uk/data-and-information/publications/statistical/hospital-admitted-patient-care-activity/2020-21)

[3. Sasieni PD, Shelton J, Ormiston-Smith N, Thomson CS, Silcocks PB. What is the lifetime risk of developing cancer?: the effect of adjusting for multiple primaries. Br J Cancer. 2011;105(3):460-5.](https://digital.nhs.uk/data-and-information/publications/statistical/hospital-admitted-patient-care-activity/2020-21)

[4. Berry JD, Dyer A, Cai X, Garside DB, Ning H, Thomas A, et al. Lifetime risks of cardiovascular disease. N Engl J Med. 2012;366(4):321-9.](https://digital.nhs.uk/data-and-information/publications/statistical/hospital-admitted-patient-care-activity/2020-21)

[5. Abu Hilal M, Besselink MG, Lemmers DHL, Taylor MA, Triboldi A. Early look at the future of healthcare during the COVID-19 pandemic. Br J Surg. 2020;107(7):e197.](https://digital.nhs.uk/data-and-information/publications/statistical/hospital-admitted-patient-care-activity/2020-21)

[6. Dobbs TD, Gibson JAG, Fowler AJ, Abbott TE, Shahid T, Torabi F, et al. Surgical activity in England and Wales during the COVID-19 pandemic: a nationwide observational cohort study. Br J Anaesth. 2021;127(2):196-204.](https://digital.nhs.uk/data-and-information/publications/statistical/hospital-admitted-patient-care-activity/2020-21)

[7. Secondary Care Analytical Team ND. Hospital Episode Statistics - Admitted Patient Care 2020-21 [Available from: https://digital.nhs.uk/data-and-information/publications/statistical/hospital-admitted-patient-care-activity/2020-21](https://digital.nhs.uk/data-and-information/publications/statistical/hospital-admitted-patient-care-activity/2020-21).

8. Office of National Statistics. Life Tables 2022 [Available from: <https://www.ons.gov.uk/peoplepopulationandcommunity/birthsdeathsandmarriages/lifeexpectancies/bulletins/nationallifetablesunitedkingdom/previousReleases>.

9. Tomic D, Morton JI, Chen L, Salim A, Gregg EW, Pavkov ME, et al. Lifetime risk, life expectancy, and years of life lost to type 2 diabetes in 23 high-income jurisdictions: a multinational, population-based study. Lancet Diabetes Endocrinol. 2022;10(11):795-803.

10. Magliano DJ, Shaw JE, Shortreed SM, Nusselder WJ, Liew D, Barr EL, et al. Lifetime risk and projected population prevalence of diabetes. Diabetologia. 2008;51(12):2179-86.

11. Schouten LJ, Straatman H, Kiemeney LA, Verbeek AL. Cancer incidence: life table risk versus cumulative risk. J Epidemiol Community Health. 1994;48(6):596-600.

12. Feigin VL, Nguyen G, Cercy K, Johnson CO, Alam T, Parmar PG, et al. Global, Regional, and Country-Specific Lifetime Risks of Stroke, 1990 and 2016. N Engl J Med. 2018;379(25):2429-37.

**SAP: Sample tables and flow chart**

**Sample table 1: Life Tables**

| **Age (years)** | Population | Incident rate of surgery | Mortality rate for those undergone surgery | Mortality rate for those who have not undergone surgery | Probability of transition from no surgery to surgery | Probability of transition from no surgery to death | Probability of transition from surgery to death | Proportion of hypothetical cohort undergoing surgery |
| --- | --- | --- | --- | --- | --- | --- | --- | --- |
| **0-4** |  |  |  |  |  |  |  |  |
| **5-9** |  |  |  |  |  |  |  |  |
| **10-14** |  |  |  |  |  |  |  |  |
| **15-19** |  |  |  |  |  |  |  |  |
| **20-24** |  |  |  |  |  |  |  |  |
| **25-29** |  |  |  |  |  |  |  |  |
| **30-34** |  |  |  |  |  |  |  |  |
| **35-39** |  |  |  |  |  |  |  |  |
| **40-44** |  |  |  |  |  |  |  |  |
| **45-49** |  |  |  |  |  |  |  |  |
| **50-54** |  |  |  |  |  |  |  |  |
| **55-59** |  |  |  |  |  |  |  |  |
| **60-64** |  |  |  |  |  |  |  |  |
| **65-69** |  |  |  |  |  |  |  |  |
| **70-74** |  |  |  |  |  |  |  |  |
| **75-79** |  |  |  |  |  |  |  |  |
| **80-84** |  |  |  |  |  |  |  |  |
| **85-89** |  |  |  |  |  |  |  |  |
| **90+** |  |  |  |  |  |  |  |  |

**Sample table 2: Incident rate**

|  | **__ to __** | | **__ to __** | |
| --- | --- | --- | --- | --- |
| **Age groups (years)** | **Number of patients undergoing surgery** | **Person-years lived** | **Number of patients undergoing surgery** | **Person-years lived** |
| **0-4** |  |  |  |  |
| **5-9** |  |  |  |  |
| **10-14** |  |  |  |  |
| **15-19** |  |  |  |  |
| **20-24** |  |  |  |  |
| **25-29** |  |  |  |  |
| **30-34** |  |  |  |  |
| **35-39** |  |  |  |  |
| **40-44** |  |  |  |  |
| **45-49** |  |  |  |  |
| **50-54** |  |  |  |  |
| **55-59** |  |  |  |  |
| **60-64** |  |  |  |  |
| **65-69** |  |  |  |  |
| **70-74** |  |  |  |  |
| **75-79** |  |  |  |  |
| **80-84** |  |  |  |  |
| **85-89** |  |  |  |  |
| **90+** |  |  |  |  |

**Sample table 3: Lifetime Risk**

|  | **Pre-Pandemic** | | **Pandemic** | |
| --- | --- | --- | --- | --- |
| **Age groups (years)** | **Lifetime risk (%)** | **95% CI** | **Lifetime risk (%)** | **95% CI** |
| **0-4** |  |  |  |  |
| **5-9** |  |  |  |  |
| **10-14** |  |  |  |  |
| **15-19** |  |  |  |  |
| **20-24** |  |  |  |  |
| **25-29** |  |  |  |  |
| **30-34** |  |  |  |  |
| **35-39** |  |  |  |  |
| **40-44** |  |  |  |  |
| **45-49** |  |  |  |  |
| **50-54** |  |  |  |  |
| **55-59** |  |  |  |  |
| **60-64** |  |  |  |  |
| **65-69** |  |  |  |  |
| **70-74** |  |  |  |  |
| **75-79** |  |  |  |  |
| **80-84** |  |  |  |  |
| **85-89** |  |  |  |  |
| **90+** |  |  |  |  |

**Sample Flow chart**

**
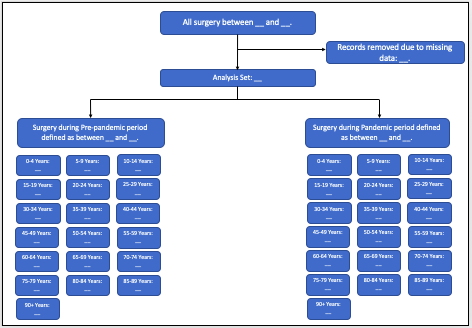
**

**Change log for SAP:**

22^nd^ December 2023

We have made the following changes for the life table method to acknowledge a new interpretation and the methods were amended to this.

We calculated lifetime risk for both men and women in a sex-differentiated fashion because the life table method does not allow combined analyses. The incident rate of surgery in person-years was calculated for each 5-year epoch. We calculated surgical mortality rate and the all-cause mortality rate in person-years for each 5-year epoch.

The probability of no longer being alive and surgery free was calculated using equation 1^4^:

$$\lambda_{i}=r_{i}+m_{i}-s_{i}$$

Where $r_{i}$ is the incident rate of surgery in person years, $m_{i}$ is the all-cause mortality rate and $s_{i}$ is the surgical mortality rate. The probability of being alive and without previous surgery at age i was calculated using equation 2^4^:

$$S_{0}(i)=exp[-5\sum_{x=5}^{i} \lambda_{i}]$$

This was multiplied by 5 due to 5-year epochs being used.

Lifetime risk at age *i* was then calculated using equation 3^4^:

$${l_{i}=S}_{0}(i)\times r_{i} \times\frac{1-exp(-{5(\lambda}_{i}))}{\lambda_{i}}$$

The lifetime risk for period *y* (e.g. from 0-90) is calculated using equation 4^4^:

$$LR(y)= \sum_{i=0}^{90} l_{i}(y)$$

**Appendix 2**

### *The Life Table Method*

We calculated lifetime risk for both men and women in a sex-differentiated fashion because the life table method does not allow combined analyses. The incident rate of surgery in person-years was calculated for each 5-year epoch. We calculated surgical mortality rate and the all-cause mortality rate in person-years for each 5-year epoch.

The probability of no longer being alive and surgery free was calculated using equation 1^4^:

$$\lambda_{i}=r_{i}+m_{i}-s_{i}$$

Where $r_{i}$ is the incident rate of surgery in person years, $m_{i}$ is the all-cause mortality rate and $s_{i}$ is the surgical mortality rate. The probability of being alive and without previous surgery at age i was calculated using equation 2^4^:

$$S_{0}(i)=exp[-5\sum_{x=5}^{i} \lambda_{i}]$$

This was multiplied by 5 due to 5-year epochs being used.

Lifetime risk at age *i* was then calculated using equation 3^4^:

$${l_{i}=S}_{0}(i)\times r_{i} \times\frac{1-exp(-{5(\lambda}_{i}))}{\lambda_{i}}$$

The lifetime risk for period *y* (e.g. from 0-90) is calculated using equation 4^4^:

$$LR(y)= \sum_{i=0}^{90} l_{i}(y)$$

*Cumulative Lifetime Incidence*

Cumulative incidence was also used to estimate the lifetime risk of surgery, it was possible to calculate the overall cumulative lifetime incidence of first and all surgery as this method does not rely on mortality rates so male and female data can be combined. We calculated this using equation 6^10^:

$$P=1-exp(-5\sum_{x=5}^{i} r_{i})$$

Where P was the cumulative incidence until age i and $r_{i}$ is the incidence rate in person-years. This was multiplied by 5 as we used 5-year epochs.

**Supplementary figure 1:** Men (blue line) compared to women (red line) cumulative lifetime incidence of first surgery based on the years 2016-19. Error bars are the 95% CIs.

**Supplementary figure 2:** Men (blue line) compared to women (red line) cumulative lifetime incidence of all surgery based on the years 2016-19. Error bars are the 95% CIs.

**Supplementary figure 3:** Pre-pandemic (blue line) compared to the pandemic (red line) lifetime risk of all surgery for men and women (Error bars are 95% CIs). The pre-pandemic period was based on data from 2016-19 and the pandemic period was based on data from 2020. This was calculated using the life table method.

**Supplementary figure 4:** Pre-pandemic (blue line) compared to the pandemic (red line) lifetime risk of first surgery for men and women (Error bars are 95% CIs). The pre-pandemic period was based on data from 2016-19 and the pandemic period was based on data from 2020. This was calculated using the life table method.
